# Supplementary material for: Mitochondria reorganization upon proliferation arrest predicts individual yeast cell fate
Source: eLife. 2018 Oct 9;7:e35685. doi: 10.7554/eLife.35685 (PMC6177259; doi:10.7554/eLife.35685)
Supplement: Supplementary file 1. [file elife-35685-supp1.docx]

| **Strains** | **References** |
| --- | --- |
| ILV3-tdimer-URA3, *his3∆1, leu2∆0, met15∆0, ura3∆0*; BY (Mata) | *Jimenez et al. J Cell Sci. 2014* |
| ILV3-GFP-HIS3, *his3∆1, leu2∆0, met15∆0, ura3∆0*; BY (Mata) | ThermoFisher Scientific |
| ILV3-GFP-HIS3, *dnm1::kanMX4, his3∆1, leu2∆0, lys2∆0, ura3∆0*; BY (Matα) | this paper |
| ILV3-GFP-HIS3, *fis1::kanMX4, his3∆1, leu2∆0, lys2∆0, ura3∆0*; BY (Matα) | this paper |
| ILV3-GFP-HIS3, *caf4::kanMX4, his3∆1, leu2∆0, lys2∆0, ura3∆0*; BY (Matα) | this paper |
| ILV3-GFP-HIS3, *mdv1::kanMX4, his3∆1, leu2∆0, lys2∆0, ura3∆0*; BY (Matα) | this paper |
| ILV3-tdimer-URA3, COX4-3xEGFP-LEU2 *his3∆1, leu2∆0, met15∆0, ura3∆0;* BY (Mata) | this paper |
| ILV3-tdimer-URA3, ABF2-GFP-HIS3, *his3∆1, leu2∆0, met15∆0, ura3∆0;* BY (Mata) | this paper |
| ILV3-tdimer-URA3, FCJ1-3xEGFP-LEU2, *his3∆1 leu2∆0, met15∆0, ura3∆0*; BY (Mata) | *Jimenez et al. J Cell Sci. 2014* |
| ILV3-GFP-HIS3; ATP14-tdimer-URA3, *his3∆1, leu2∆0, met15∆0, ura3∆0;* BY (Mata) | *Jimenez et al. J Cell Sci. 2014* |
| ABP1-3xGFP-URA3, *his3∆1, leu2∆0, met15∆0, ura3∆0;* BY (Mata) | *Sagot et al. Mol Biol Cell. 2006* |
| PRE5-GFP-HIS3, *his3∆1, leu2∆0, met15∆0, ura3∆0*; BY (Mata) | ThermoFisher Scientific |
| ABP1-GFP-HIS3, ILV3-tdimer-URA3, *his3∆1, leu2∆0, met15∆0, ura3∆0;* BY (Mata) | this paper |
| ILV3-tdimer-URA3, Scl1-GFP-HIS3; *his3∆1, leu2∆0, met15∆0, ura3∆0*; BY (Mata) | this paper |
| TOM20-GFP-HIS3, *his3∆1, leu2∆0, met15∆0, ura3∆0;* BY (Mata) | ThermoFisher Scientific |
| NDI1-GFP-HIS3, *his3∆1, leu2∆0, met15∆0, ura3∆0;* BY (Mata) | ThermoFisher Scientific |
| ALD5-GFP-HIS3, *his3∆1, leu2∆0, met15∆0, ura3∆0;* BY (Mata) | ThermoFisher Scientific |
| ILV3-tdimer-URA3; ATP3-GFP-HIS3, *his3∆1, leu2∆0, met15∆0, ura3∆0;* BY (Mata) | *Jimenez et al. J Cell Sci. 2014* |
| COX7-GFP-HIS3, ILV3-tdimer-URA3, *his3∆1, leu2∆0, met15∆0, ura3∆0;* BY (Mata) | *Jimenez et al. J Cell Sci. 2014* |
| POR1-GFP-HIS3, *his3∆1, leu2∆0, met15∆0, ura3∆0;* BY (Mata) | ThermoFisher Scientific |
| TOM70-GFP-HIS3, *his3∆1, leu2∆0, met15∆0, ura3∆0;* BY (Mata) | ThermoFisher Scientific |
| QCR9-GFP-HIS3, *his3∆1, leu2∆0, met15∆0, ura3∆0;* BY (Mata) | ThermoFisher Scientific |
| TIM23-GFP-HIS3, *his3∆1, leu2∆0, met15∆0, ura3∆0;* BY (Mata) | ThermoFisher Scientific |
| QCR2-GFP-HIS3, *his3∆1, leu2∆0, met15∆0, ura3∆0;* BY (Mata) | ThermoFisher Scientific |
| PAM17-GFP-HIS3, *his3∆1, leu2∆0, met15∆0, ura3∆0;* BY (Mata) | ThermoFisher Scientific |
| TIM44-GFP-HIS3, *his3∆1, leu2∆0, met15∆0, ura3∆0;* BY (Mata) | ThermoFisher Scientific |
| COX6-GFP-HIS3, *his3∆1, leu2∆0, met15∆0, ura3∆0;* BY (Mata) | ThermoFisher Scientific |
| OXA1-GFP-HIS3, *his3∆1, leu2∆0, met15∆0, ura3∆0;* BY (Mata) | ThermoFisher Scientific |
| TIM50-GFP-HIS3, *his3∆1, leu2∆0, met15∆0, ura3∆0;* BY (Mata) | ThermoFisher Scientific |
| PRX1-GFP-HIS3, *his3∆1, leu2∆0, met15∆0, ura3∆0;* BY (Mata) | ThermoFisher Scientific |
| SOD2-GFP-HIS3, *his3∆1, leu2∆0, met15∆0, ura3∆0;* BY (Mata) | ThermoFisher Scientific |
| FMP16-GFP-HIS3, *his3∆1, leu2∆0, met15∆0, ura3∆0;* BY (Mata) | ThermoFisher Scientific |
| MDH1-GFP-HIS3, *his3∆1, leu2∆0, met15∆0, ura3∆0;* BY (Mata) | ThermoFisher Scientific |
| PDB1-GFP-HIS3, *his3∆1, leu2∆0, met15∆0, ura3∆0;* BY (Mata) | ThermoFisher Scientific |
| NDE1-GFP-HIS3, *his3∆1, leu2∆0, met15∆0, ura3∆0;* BY (Mata) | ThermoFisher Scientific |
| YHM2-GFP-HIS3, *his3∆1, leu2∆0, met15∆0, ura3∆0;* BY (Mata) | ThermoFisher Scientific |
| MNP1-GFP-HIS3, *his3∆1, leu2∆0, met15∆0, ura3∆0;* BY (Mata) | ThermoFisher Scientific |
| RIM1-GFP-HIS3, *his3∆1, leu2∆0, met15∆0, ura3∆0;* BY (Mata) | ThermoFisher Scientific |
| AIM37-GFP-HIS3, *his3∆1, leu2∆0, met15∆0, ura3∆0;* BY (Mata)) | ThermoFisher Scientific |
| AIM5-GFP-HIS3, *his3∆1, leu2∆0, met15∆0, ura3∆0;* BY (Mata) | ThermoFisher Scientific |
| OM45-GFP-HIS3, *his3∆1, leu2∆0, met15∆0, ura3∆0;* BY (Mata) | ThermoFisher Scientific |
| WT ; FY4 (a) | F. Winston |
| Rho0 ; FY4 (a) | This paperNA |
| ILV3-tdimer-URA3, *his3∆1, leu2∆0, met15∆0, ura3∆0;* BY (Mata) Rho0 | this paper |
| mmm1::kanMX4; ILV3-tdimer-URA3; *his3∆1, leu2∆0, lys2∆0, ura3∆0;* BY (Matα) | this paper |
| ILV3-tdimer-URA3, *ura3-1, trp1-1, ade2-1, leu2-3,112, his3-11,15*, W303 (Mata) | this paper |
| ILV3-tdimer-URA3, *ura3-52, trp1-289, leu2-3,112, his3∆1, MAL2-8c, SUC2,* CENPK (Mata) | this paper |
| hst4::KanMX4, *his3∆1, leu2∆0, lys2∆0, ura3∆0;* BY (Matα) | GE Healthcare Dharmacon Inc. |
| hda2::KanMX4, *his3∆1, leu2∆0, lys2∆0, ura3∆0;* BY (Matα) | GE Healthcare Dharmacon Inc. |
| rpl35B::KanRMX4, *his3∆1, leu2∆0, lys2∆0, ura3∆0;* BY (Matα) | GE Healthcare Dharmacon Inc. |
| rpd3::KanRMX4, *his3∆1, leu2∆0, lys2∆0, ura3∆0;* BY (Matα) | GE Healthcare Dharmacon Inc. |
| whi5::KanRMX4, *his3∆1, leu2∆0, lys2∆0, ura3∆0;* BY (Matα) | GE Healthcare Dharmacon Inc. |
| sir2::KanRMX4, *his3∆1, leu2∆0, lys2∆0, ura3∆0;* BY (Matα) | GE Healthcare Dharmacon Inc. |
| hst3::KanRMX4, *his3∆1, leu2∆0, lys2∆0, ura3∆0;* BY (Matα) | GE Healthcare Dharmacon Inc. |
| cln3::KanRMX4, *his3∆1, leu2∆0, lys2∆0, ura3∆0;* BY (Matα) | GE Healthcare Dharmacon Inc. |
| xrn1::KanRMX4, *his3∆1, leu2∆0, lys2∆0, ura3∆0;* BY (Matα) | GE Healthcare Dharmacon Inc. |
| ILV3-tdimer-URA3; SCL1-GFP-HIS3; *his3∆1, leu2∆0, met15∆0, ura3∆0;* BY (Mata) Rho0 | this paper |
| ILV3-tdimer-URA3, *fis1::kanMX4, ura3-1, trp1-1, ade2-1, leu2-3,112, his3-11,15* W303 (Mata) | this paper |
| atg32::KanR; ILV3-tdimer-URA3, *ura3-1, trp1-1, ade2-1, leu2-3,112, his3-11,15,* W303 (Mata) | this paper |
